# A step-by-step diagnosis of exclusion in a twin pregnancy with acute respiratory failure due to non-fatal amniotic fluid embolism: a case report

**DOI:** 10.1186/1752-1947-2-177

**Published:** 2008-05-27

**Authors:** Vasilios E Papaioannou, Christos Dragoumanis, Vassiliki Theodorou, Dimitrios Konstantonis, Ioannis Pneumatikos

**Affiliations:** 1 Department of Intensive Care Medicine, Alexandroupolis University Hospital, Democritus University of Thrace, Medical School, Dragana, Alexandroupolis 68100, Greece

## Abstract

**Introduction:**

Respiratory failure may develop during the later stages of pregnancy and is usually associated with tocolysis or other co-existing conditions such as pneumonia, sepsis, pre-eclampsia or amniotic fluid embolism syndrome.

**Case presentation:**

We present the case of a 34-year-old healthy woman with a twin pregnancy at 31 weeks and 6 days who experienced acute respiratory failure, a few hours after administration of tocolysis (ritodrine), due to preterm premature rupture of the membranes. Her chest discomfort was significantly ameliorated after the ritodrine infusion was stopped and a Cesarean section was performed 48 hours later under spinal anesthesia; however, 2 hours after surgery she developed severe hypoxemia, hypotension, fever and mild coagulopathy. The patient was intubated and transferred to the intensive care unit where she made a quick and uneventful recovery within 3 days. As there was no evidence for drug- or infection-related thromboembolic or myocardial causes of respiratory failure, we conclude that our patient experienced a rare type of non-fatal amniotic fluid embolism.

**Conclusion:**

In spite of the lack of solid scientific support for our diagnosis, we conclude that our patient suffered an uncommon type of amniotic fluid embolism syndrome and we believe that this report highlights the need for extreme vigilance and a high index of suspicion for such a diagnosis in any pregnant individual.

## Introduction

Mild dyspnea is a common symptom during late pregnancy. However, some women experience severe respiratory distress before or immediately after labor. This could be the result of co-existing conditions, such as asthma or cardiovascular disease. Others may have an acute illness such as pneumonia, pneumothorax or pulmonary embolism. Finally, some pregnancies are complicated by pulmonary edema of cardiac or non-cardiac origin. In a previously healthy woman the first case is usually a drug-related complication (mainly due to tocolysis). The latter could be secondary to increased permeability of the pulmonary vasculature, due to pre-eclampsia, septic shock, placental abruption, major obstetric hemorrhage and amniotic fluid embolism (AFE) syndrome [1].

We present a case of a previously healthy woman with a twin pregnancy who at 31 weeks and 6 days experienced a biphasic pattern of respiratory distress and pulmonary edema, fever and coagulopathy, premature rupture of the membranes (PROM) and use of tocolysis initially after preterm labor and, subsequently, shortly after delivery. By step-by-step exclusion of every possible cause of acute lung injury, we concluded that this is a rare case of acute respiratory failure due to AFE in a twin pregnancy.

## Case presentation

A 34-year-old healthy woman with a twin pregnancy at 31 weeks and 6 days was admitted to our hospital with premature uterine contractions. She had no history of previous pregnancy, allergy or smoking. Vaginal examination revealed the presence of pooled amniotic fluid on a sterile speculum. Preterm PROM was diagnosed and a ritodrine infusion was started at a dose of 0.10 to 0.3 mg/minute, given in 1000 ml of normal saline, for 24 hours. At 24 hours, uterine contractions were arrested successfully, the ritodrine infusion was tapered and oral ritodrine was begun with 2 mg every 2 hours. She was also given dexamethasone (two 12 mg doses) to improve fetal lung maturation. Over the next 24 hours she became increasingly breathless with a tachycardia of 140 beats/minute, blood pressure of 110/70 mmHg, bilateral basal crackles and temperature of 37.6°C. Cardiotocography (CTG) revealed no signs of fetal distress. Pulmonary edema was diagnosed clinically and ritodrine administration was stopped, while she responded to a bolus of intravenous furosemide. Antibiotic treatment (amoxicillin/clavulanic acid 1000 mg/100 mg four times a day intravenously and erythromycin 1 g four times a day intravenously) was started to prevent possible intrauterine infection and nadroparin calcium (2850 IU once daily subcutaneously) was added for venous thomboprophylaxis. On the suspicion of an intrauterine infection an uneventful Cesarean section was performed 48 hours later, under spinal anesthesia, and the patient delivered healthy twins (Apgar score: 9 and 8 at 1 minute and 10 at 5 minutes for both neonates). As Cesarean section requires a T4 sensory level, 1.5 liters of normal saline was administered intravenously prior to surgery and 1 liter during surgery.

**Figure 1:**
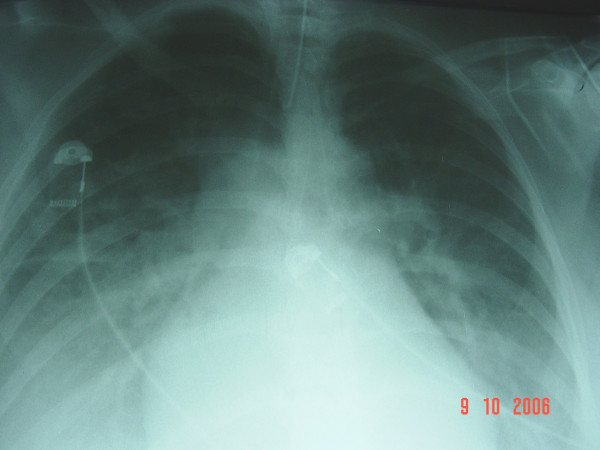
Acute bilateral pulmonary edema with pleural effusions.

A few hours after delivery the patient became acutely dyspnoeic with a respiratory rate of 35 breaths/minute and bilateral rhonchi. Blood pressure was 75/45 mmHg. The electrocardiogram showed a sinus tachycardia of 123 beats/minute. In spite of treatment with oxygen via nasal spectacles (15 liters/minute), her arterial blood gas analysis showed a severe hypoxemia with cyanosis (pH 7.46, PaO_2 _6.25 kPa, PaCO_2 _3.99, bicarbonate 22 mmol/L). The patient was intubated and transferred to the intensive care unit (ICU). A chest X-ray (Figure 1) revealed bilateral pulmonary edema with pleural effusions while a spiral computed tomography (CT) scan of the thorax supported the above findings and excluded any case of pulmonary embolism. A noradrenaline infusion was started at a low rate (2 μg/minute) during initial resuscitation to support blood pressure; noradrenaline infusion was gradually reduced and stopped after 90 minutes as the patient's hemodynamics stabilized. Central venous pressure was 12 mmHg under mechanical ventilatory support. An echocardiogram showed good biventricular function with normal chamber dimensions while there was no elevation in cardiac enzymes. Duplex ultrasound scanning of the lower extremities revealed no thrombosis of the femoral and popliteal veins.

During her first day in the ICU the patient developed fever (38.8°C), leucocytosis (17 × 10^9^/liter) and mild coagulopathy (platelets 110 × 10^9^/liter, activated partial thromboplastin time 47 seconds, fibrinogen 140 mg/dl). A serologic examination of pleural fluid was performed and revealed no signs of exudate. Extensive cultures (blood, sputum and vagina) remained negative, while C-reactive protein (CRP) was increased (15 mg/dl). After the third day of treatment, the patient made a quick recovery with complete resolution of the pulmonary edema and she was extubated 1 day later.

## Discussion

Our patient matched well with the Clark criteria for AFE: hypotension, pulmonary edema, cyanosis, coagulopathy, dyspnea. However, the presence of PROM and prior ritodrine toxicity complicated the clinical picture [2]. PROM is defined as rupture of the chorioamniotic membranes before the onset of labor. Maternal complications with preterm PROM are more common, with chorioamnionitis rates approximating 25% to 35% (see [3,4]).

Suppression of uterine contractions seems to be the obvious solution to the problem of preterm labor. Our patient received ritodrine for tocolysis, which is a β_2 _sympathomimetic agent and has been clearly shown to prolong pregnancy by 48 hours. There is a strong association between its use and the development of maternal pulmonary edema, especially with concomitant administration of steroids. This complication has been reported in up to 9% of cases and has been responsible for at least 15 maternal deaths [5,6]. The initial tachycardia and tachypnea of the patient was attributed to the ritodrine infusion, so the drug was discontinued. Her clinical status was significantly improved; however, antibiotics were administered due to the risk of intrauterine infection.

Two days later and approximately 2 hours after the scheduled Cesarean section, the patient developed acute respiratory distress and was transferred to the ICU. Acute myocardial infarction was not supported by typical electrocardiographic and echocardiographic changes and elevated enzymes. Duplex scanning of both extremities showed the absence of thrombosis, which made deep venous thrombosis unlikely. The normal CT scanning findings made pulmonary embolism less probable. Tocolytic therapy was also an unlikely cause because ritodrine, with an elimination half-life of 1 to 3 hours [7], was discontinued approximately 48 hours before the onset of acute respiratory failure.

High spinal anesthesia associated with vasomotor block, profound bradycardia and respiratory insufficiency could be responsible for the postoperative hypotension and tachypnea. In cases such as a twin pregnancy, the gravid uterus increases intra-abdominal pressure significantly and decreases the epidural and subarachnoid space by the associated engorgement of the epidural venus plexus. In these circumstances, the usual recommended dose of spinal anesthesia for non-pregnant patients will have a more cephalad spread and may cause significant maternal hypotension and even hypoperfusion of the medullary respiratory center [8]. However, in our case we administered a much lower dose of local anesthetic (8 mg of 0.75% ropivacaine), the level of sensory anesthesia never extended above T4 and the patient was well hydrated prior to and during surgery.

Clinical chorioamnionitis was not supported from clinical examination (there was no uterine tenderness, purulent vaginal discharge or fetal tachycardia), while pneumonia, sepsis or septic shock were unlikely causes of respiratory failure, as there was no positive culture and the rapid resolution of pulmonary edema did not support their diagnosis. However, alterations in cellular immunity due to hormones prevalent during pregnancy, such as progesterone and human chorionic gonadotropine, the history of preterm PROM and the administration of tocolysis that is associated with the development of pneumonia, could not rule out a subclinical infection completely [9,10]. CRP was increased but this could be due to any inflammatory process without concomitant infection. Radiological findings were not specific for pneumonia, but even in patients with symptoms consistent with a lower respiratory tract infection, radiologically proven pneumonia is confirmed in only 39% of cases [9]. Furthermore, biochemical analysis of the pleural fluid did not reveal signs of exudate.

The final diagnosis that was made, therefore, by exclusion of other causes of respiratory distress and pulmonary edema, was a case of AFE syndrome. AFE occurs in about 1:40,000 to 1:60,000 deliveries and has a mortality rate of over 85%. AFE appears to be initiated after maternal intravascular exposure to fetal tissues and usually occurs during labor, but may occur also as early as the 20th gestational week or as late as 32 hours post-partum. Patients present mainly with a sudden collapse associated with dyspnea, cyanosis and hypotension [2]. Those surviving the initial phase develop pulmonary edema (75%).

It has been suggested that AFE is clinically, hemodynamically and hematologically indistinguishable from anaphylaxis and septic shock [2]. There is no definite clinical or laboratory diagnosis, except for necropsy that demonstrates fetal squamous cells, mucin, hair or vernix in the pulmonary vasculature. The diagnosis therefore is made by exclusion of other causes with similar clinical findings [11,12]. The hematological, pulmonary or hemodynamic alterations can vary in presentation or can be entirely absent. Respiratory distress is found to predominate in 51% of patients, hypotension in 27% and coagulopathy in 12% (see [12]). The pathophysiology of AFE is not completely understood. Although AFE in the past was attributed to mechanical obstruction of the pulmonary vessels by amniotic fluid, at present the endothelial injury from the biologically active substances tissue factor, endothelin, histamin, prostaglandins and complement activation in the amniotic fluid seems a more likely explanation for the pathogenesis of AFE [12].

The occurrence of AFE in twin pregnancy is extremely rare. To the best of the authors' knowledge, only four cases have been described in the literature [10,13-15]. We consider this case to represent an uncommon type of AFE with severe respiratory distress, mild hypotension, fever and mild coagulopathy, despite the absence of any known risk factors such as tumultuous labor, use of uterine stimulants, advanced maternal age or meconium in the amniotic fluid [12]. Although histology of the placenta was not performed in order to definitely exclude a case of chorioamnionitis, and pulmonary or pleural fluid was not analyzed for the presence of lanugo or squames, because AFE was unfortunately not considered as a possible diagnosis, we believe that this is a case of mild acute respiratory failure due to pulmonary AFE syndrome. This highlights the need for extreme vigilance and a high index of suspicion for AFE by the attending physician in a case of any post-partum individual with respiratory failure, especially in the presence of risk factors such elderly primigravida, multipara and instrumental delivery [16].

## Conclusion

Pulmonary edema may develop in pregnancy, especially in the later stages, either as a tocolysis-related complication or due to increased permeability of the pulmonary vasculature, due to pre-eclampsia, septic shock or AFE syndrome. Our patient developed a biphasic pattern of acute respiratory distress, with initial chest discomfort and tachypnea that were attributed to, after excluding other possible causes, ritodrine administration due to preterm PROM, and subsequently with severe hypoxemia, hypotension and mild coagulopathy following Cesarean section that were associated with a rare type of non-fatal AFE.

Differential diagnosis of severe respiratory distress in such patients may be extremely difficult since common symptoms and signs of sepsis, pneumonia, thromboembolism and acute heart failure sometimes lack sensitivity and specificity, whereas regional anesthetic techniques that are usually implemented for urgent Cesarean section may further complicate the clinical picture.

AFE remains more or less a diagnosis of exclusion and despite its severe clinical appearance it can be manifested as a more subtle form of respiratory failure and cardiovascular compromise. We believe that despite the lack of specific scientific evidence to support our diagnosis, this case represents an uncommon type of non-fatal AFE and physicians responsible for the care of a high-risk pregnancy should be familiar with its clinical course.

## Abbreviations

AFE: amniotic fluid embolism; CRP: C-reactive protein; CT: computed tomography; ICU: intensive care unit; PROM: premature rupture of the membranes.

## Competing interests

The authors declare that they have no competing interests.

## Consent

Written informed consent was obtained from the patient for publication of this case report and any accompanying images. A copy of the written consent is available for review by the Editor-in-Chief of this journal.

## Authors' contributions

PV conceived of the idea for the publication, performed the literature search and was the principal writer of the manuscript, CD helped to draft the manuscript and assisted with the collection of biomedical data, VT and DK helped with the collection of biomedical data, IP critically revised the manuscript and gave final approval of the version to be published.
